# Microvesicles from Mesenchymal Stromal Cells Are Involved in HPC-Microenvironment Crosstalk in Myelodysplastic Patients

**DOI:** 10.1371/journal.pone.0146722

**Published:** 2016-02-02

**Authors:** Sandra Muntión, Teresa L. Ramos, María Diez-Campelo, Beatriz Rosón, Luis Ignacio Sánchez-Abarca, Irena Misiewicz-Krzeminska, Silvia Preciado, María-Eugenia Sarasquete, Javier de las Rivas, Marcos González, Fermín Sánchez-Guijo, María-Consuelo del Cañizo

**Affiliations:** 1 Servicio de Hematología, Hospital Universitario de Salamanca, Salamanca, Spain; 2 Centro en Red de Medicina Regenerativa y Terapia Celular de Castilla y León, Salamanca, Spain; 3 Red Nacional de Terapia Celular (TerCel), Instituto Nacional de Salud Carlos III, Madrid, Spain; 4 Centro de Investigación del Cáncer-IBMCC (Universidad de Salamanca-CSIC), Salamanca, Spain; 5 IBSAL-Hospital Universitario Salamanca, Salamanca, Spain; 6 National Medicines Institute, Warsaw, Poland; RWTH Aachen University Medical School, GERMANY

## Abstract

Exosomes/microvesicles (MVs) provide a mechanism of intercellular communication. Our hypothesis was that mesenchymal stromal cells (MSC) from myelodysplastic syndrome (MDS) patients could modify CD34^+^ cells properties by MVs. They were isolated from MSC from MDS patients and healthy donors (HD). MVs from 30 low-risk MDS patients and 27 HD were purified by ExoQuick-TC™ or ultracentrifugation and identified by transmission electron microscopy, flow cytometry (FC) and western blot for CD63. Incorporation of MVs into CD34^+^ cells was analyzed by FC, and confocal and fluorescence microscopy. Changes in hematopoietic progenitor cell (HPC) properties were assessed from modifications in microRNAs and gene expression in CD34^+^ cells as well as viability and clonogenic assays of CD34^+^ cells after MVs incorporation. Some microRNAs were overexpressed in MVs from patients MSC and two of them, miR-10a and miR-15a, were confirmed by RT-PCR. These microRNAs were transferred to CD34^+^ cells, modifying the expression of *MDM2* and *P53* genes, which was evaluated by RT-PCR and western blot. Finally, examining CD34^+^ cells properties after incorporation, higher cell viability (p = 0.025) and clonogenic capacity (p = 0.037) were observed when MVs from MDS patients were incorporated. In summary, we show that BM-MSC release MVs with a different cargo in MDS patients compared with HD. These structures are incorporated into HPC and modify their properties.

## Introduction

Myelodysplastic syndromes (MDS) constitute a heterogeneous group of clonal hematological disorders characterized by the presence of peripheral cytopenias and an increased risk of transformation into acute myeloblastic leukemia (AML)[1, 2]. The pathophysiology of these disorders is complex but their origin in a clonal hematopoietic stem cell disorder is fully accepted. Many genomic aberrations and abnormalities in the microRNAs expression profile in hematopoietic progenitor cells (HPC) are involved in the development of MDS, as a very important mechanism[3].

Finally, in the last few years, the importance of the bone marrow (BM) microenvironment has been highlighted[4].

Mesenchymal stromal cells (MSC) are a non-hematopoietic BM cell population considered to be the osteoblastic progenitors and a key component of the hematopoietic microenvironment. Our group[5] and others[6–8] have shown that MSC exhibit several morphological, functional and genetic alterations in MDS patients. In this regard, Raaijmakers et al.[9] have recently demonstrated in a murine model that the deletion of *DICER*, an RNase III enzyme involved in microRNA biogenesis, in MSC-derived osteoprogenitors not only affected their differentiation but also resulted in peripheral blood cytopenias, myelodysplasia and secondary leukemia, providing evidence that specific molecular alterations in the bone marrow microenvironment could result in clonally impaired hematopoiesis. We have also shown that MSC from MDS patients, compared with healthy subjects, have a lower level of expression of *DICER1* and *DROSHA*[10].

Intercellular communication can be achieved by direct cell-to-cell contact or the exchange of soluble factors. A new mechanism of intercellular communication based on the secretion of exosomes/microvesicles has been described. Such a mechanism modifies the functional properties of recipient cells by the transfer of bioactive molecules such as mRNA, proteins and microRNAs, among others[11]. The vesicles are a mixed population of exosomes and shedding vesicles and both components, despite originating from different cellular structures, participate in the communication between the microenvironment and the HPC. In this manuscript we will refer to them as microvesicles (MVs).

It has been suggested that MVs derived from human bone marrow MSC may act as mediators of cell-to-cell communication through microRNAs delivery[12]. These transferred microRNAs have a function in the hematopoietic system[13].

The hypothesis of our study is that MSC from MDS patients (MDS-MSC) can modify the properties of HPC through release of MVs with a different microRNAs content as compared to MSC from healthy donors (HD-MSC).

## Methods

### Patients and control samples

Microvesicles were isolated from third-passage BM-MSC from 30 consecutive patients (median age 72 years; range 44–92 years) with "de novo" low-risk MDS at diagnosis. In order to have less variability in our sample patients only low risk cases were included. Studies were performed in each case according to the availability of material for a specific analysis. These data are shown in S1 Table.

The most important characteristics of MDS patients are described in Table 1. They were classified according to the 2008 WHO classification criteria. Male to female ratio was 21:9 and median age was 72 years (range 44–92). MVs from MSC obtained from BM samples of 27 healthy volunteer donors (16 males and 11 females) with a median age of 40 years (range 21–65 years) were used as controls. In all cases written informed consent was previously obtained according to institutional guidelines in accordance with the local Ethics Committee of the "Area de Salud de Salamanca". All experimental procedures were also approved by Ethics Committee, Hospital Universitario de Salamanca (2008/04/14).

**Table 1:** MDS patient characteristics.

Patient number	WHO 2008 classification	Conventional Cytogenetics analysis or FISH*
1	RCMD	46,XY[21]
2	RCMD	46,XY[22]
3	MDS del(5q)	78% 5q-*
4	MDS del(5q)	75% 5q-*
5	MDS del(5q)	46,XX,del(5)(q13q31)[14]/46,XX[6]
6	RARS	46,XY[20]
7	MDS del(5q)	46,XY,del(5)(q13q31)[17]/46,XY[3]
8	RCMD	46,XX[23]
9	RCUD	46,XY[23]
10	RCMD	46,XY[23]
11	RCMD	46,XX[20]
12	RCMD-RS	46,XY[23]
13	RCUD	No Mitosis
14	RCMD	46,XY[20]
15	RCMD	46,XY[20]
16	RCMD	46,XY[20]
17	RCMD	46,XY[20]
18	RCMD	46,XX[23]
19	AREB-1	46,XY[20]
20	RCMD	46,XY[25]
21	RCMD	46,XX,t(6;11)(q22;q13)[25]
22	RCMD	46,XX[24]
23	RA	47,XY,+8[4]/46,XY[6]
24	RCUD	46,XX[24]
25	RCMD	46,XY[20]
26	MDS del (5q)	71% 5q-*
27	RCMD	46,XY,del(20)(q12)[20]
28	RCMD-RS	No Mitosis
29	RCMD	46,XY[25]
30	RCMD	46,XY[22]

* For FISH analysis a minimum of 200 nuclei were analyzed. FISH: Fluorescent In Situ Hybridization; RCMD: refractory cytopenia with multilineage-dysplasia; MDS del(5q): Myelodysplastic syndrome with isolated 5q deletion; RA: refractory anemia; RARS: refractory anemia with ringed sideroblasts; RCUD: refractory cytopenia with unilineage dysplasia; RCMD-RS: RCDM with ringed sideroblasts; RAEB-1: Refractory anemia with excess of blasts

### Isolation and characterization of MSC

BM mononuclear cells (MNC) were isolated by Ficoll-Paque density-gradient centrifugation (Ficoll-Paque, density:1.077k, GE Healthcare BioSciences, Buckinghamshire, UK). BM-MNC were counted and plated at a density of 1x10^6^ cells/cm^2^ and expansion was carried out according to the previously described method[5]. After the third passage, MSC were assessed in accordance with the minimal definition criteria proposed by the International Society for Cellular Therapy (ISCT)[14], which includes the capacity to differentiate into osteoblasts, adipocytes and chondrocytes, and standard immunophenotypic analytical procedures, as previously reported[5]. Viability studies were done by flow cytometry using APC H7 Annexin V DY634 (Immunostep #ANXVDY, Salamanca, Spain).

### CD34^+^ cells isolation

Mobilized CD34^+^ progenitor cells were isolated from leukapheresis samples from 16 HD (with a male/female ratio of 10/6, median age 50 years; range 20–69 years), for allogeneic HPC transplantation, as previously described[15]. CD34^+^ progenitor cells were sorted by magnetic labeling using the human CD34 MicroBead Kit according to the manufacturer’s recommendations (Miltenyi Biotec GmbH, BergischGladbach, Germany). After isolation, the purity and viability of CD34^+^ cells was evaluated by flow cytometry using fluorescein isothiocyanate (FITC)-conjugated CD34 (11-0349-42, eBioscience Inc. San Diego, CA, USA) and APC H7 Annexin V DY634.

### MVs isolation and characterization

For MVs production, MSC from both groups (MDS and controls) at third passage were cultured in DMEM deprived of FCS. The supernatants were collected initially after 6 hours of FCS starvation (n = 3) and in the remaining cases (n = 59) after 24 hours of starvation, since the quantity of MVs obtained was higher in the latter situation.

In 22 samples, MVs were obtained using the ExoQuick-TC^TM^ methods. In 40 experiments MVs were also obtained by ultracentrifugation assay. The rationale for using both methods was initially to compare them in all the experiments performed in the study. Nevertheless, we did not find any differences between both well-established methods.

Briefly, the supernatants were initially centrifuged at 2,000 g for 20 min followed by a second centrifugation at 10,000 g for 30 min to remove debris and apoptotic bodies. Supernatants were then ultracentrifuged at 100,000 g for 70 min at 4°C in a fixed-angle rotor[16]. After that, the protein content of MVs was quantified by the Bradford method (BioRad, Hercules, CA). The size distribution of MVs from MSC was analyzed using a NanoSight LM10 instrument (NanoSight Ltd., Amesbury, UK) equipped with the nanoparticle tracking analyses (NTA) 2.0 analytic software.

#### Transmission electron microscopy

The MVs containing pellet obtained by ultracentrifugation were resuspended in 50μL of 2% paraformaldehyde, loaded on to Formvar/carbon-coated EM grids, and post-fixed in 1% glutaraldehyde. The samples were contrasted with uranyloxalate solution and examined under a transmission electron microscope (FEITecnai G2 Spirit Biotwin) using a digital camera (Morada, Soft Imaging System, Olympus).

#### Flow cytometry

To characterize MVs the following panel of monoclonal antibodies (MoAbs) was used: mouse anti-human CD90 FITC (Cat.555595), mouse anti-human CD73 PE (phycoerythrin) (Cat.550257), mouse anti-human CD63 PE (Cat.557305), mouse anti-human CD34 PerCP-Cy5.5 (phycoerythrin-cyanine 5.5) (Cat.347222), mouse anti-human CD81 APCH7 (APC-cyanine tandem dye) (Cat.656647), mouse anti-human CD45 V500 (BD Horizon V500) (Cat.560779) purchased from BD Biosciences (San Jose, CA, USA). Anti-human CD44 APC (Cat.44A2-100T) and anti-human CD105 APC (FAB10971A) were purchased from Immunostep (Salamanca, Spain) and R&D Systems (Minneapolis, MN, USA), respectively.

Before MVs acquisition we always acquired double-filtered PBS that contained the megamix of standard beads based on 1 μm monodisperse polystyrene (Sigma-Aldrich) and Perfect-Count Microspheres (Cytognos;6.0–6.4 μm) [17]. This procedure allowed us to define the threshold level and also to use as an instrument quality control for background noise. The beads of different sizes were used as size markers, and analysis was performed using a log scale for forward (FSC) and side scatter (SSC) parameters. MVs acquisition from the different samples was only processed when the number of events acquired was between 25–50 events per second at low speed, with a threshold between 300–400. For flow cytometer calibration Rainbow Calibration Kit and Rainbow QC Kit (Spherotech, Inc.) were used. These particles are known as compensation beads (CompBeads), which were also used to determine the electronic noise or background. Ranges were verified using CompBeads labeled with specific fluorochromes. Samples were acquired after the cytometer was calibrated. MVs recovered from ultracentrifugation were resuspended in double-filtered PBS and stained by direct immunofluorescence using the aforementioned MoAb panel. A total of 200,000 events were acquired in a three-laser FACS Canto II (BD Biosciences) using FACS Diva 6.1.1 Software (BD Biosciences). Data were analyzed using Infinicyt software (Cytognos)(S1 Fig).

#### Western blot analysis

MVs were lysed at 4°C for 30 min in 1X RIPA lysis buffer (1X TBS, 1% Nonidet P-40, 0.5% sodium deoxycholate, 0.1% SDS, 0.004% sodium azide) plus PMSF, protease inhibitor cocktail and sodium orthovanadate. Samples were loaded onto a 12% SDS-PAGE gradient under reducing conditions and electroblotted onto nitrocellulose membrane filters as previously described[10]. The blots were blocked with 5% non-fat milk in 20 mM Tris-HCl pH 7.5, 500 mM NaCl plus 0.1% Tween (TBS-T). MVs membranes were subsequently immunoblotted at 4°C with the appropriate primary antibody rabbit anti-human CD63 (1:1000 from System Biosciences (Catalog# EXOAB-CD63A-1)) overnight. Membranes were incubated with anti-rabbit IgG horseradish peroxidase-conjugated secondary antibody for 1hour at room temperature (Amersham Biosciences). Specific bands were visualized using ECL Western Blotting Detection Reagents (Amersham Biosciences, Mountain View, CA).

### MicroRNA from MVs expression analysis

MVs obtained from ultracentrifugation were resuspended in 500 μl Trizol (Roche Diagnostics GmbH, Mannheim, Germany) and total RNA with conservation of small RNAs was isolated using a Qiagen miRNeasy Kit. To study the microRNAs content of MSC-MVs of patients and controls the differential expression of microRNAs was analyzed as previously described[10, 18, 19]. Total RNA (<350 ng) from MSC-derived MVs of 8 low-risk MDS and 4 HD was retrotranscribed with a Megaplex™ RT Primer pool (Applied Biosystems 4399966, Foster City, CA, USA). Diluted RT reaction product is mixed with TaqMan Universal PCR Mastermix (no AmpErase UNG, 4364341) and loaded into the corresponding TaqMan low-density arrays fill ports (Applied Biosystems, part number: 4384792). This panel contains 384 PCR assays enabling accurate quantification of 378 human microRNAs and three endogenous controls (RNU44, RNU48 and 4 replicates of RNU6B) to aid in data normalization. Real-time PCR was carried out using an Applied Biosystems 7900 HT Fast Real-time PCR sequence detection system. The reactions were incubated at 94.5°C for 10 min, followed by 50 cycles of 97°C for 30s and 59.7°C for 1 min.

MicroRNAs expression data were processed within the R statistical computing environment (version 2.13.0), using ΔΔCt standard procedures from the ‘HTqPCR’ package[18]. Each microRNA raw Ct value was tagged as undetermined when fell between levels of 36 and 40. Raw Ct values were normalized using the array endogenous control features according to the equation: ΔCt_microRNA_ = Ct_microRNA_—mean (Ct _RNU6B, RNU48_). An analysis of the behavior and reliability of these controls, and an alternative ΔCt normalization protocol, was also performed in order to check for potential biases introduced by these endogenous controls (added as S1 Methods). Differences between groups were calculated based on the ΔΔCt measure, where ΔΔCt_microRNA_ = mean(ΔCt_MDS_)—mean(ΔCt_HealthyDonors_). Statistical significance was assessed using unpaired samples t-tests. Fold changes as relative quantifications of expression (i.e. FC = 2^-ΔΔCt^) for each microRNA were reported. Also each microRNA log10(FC) was added for an intuitive interpretation of change direction. Medians of raw Ct values per condition over the threshold 36 were considered qPCR undetermined measures. The GEO entry for each sample includes this information, as in the sample HD-10306, GSM1262596: http://www.ncbi.nlm.nih.gov/geo/query/acc.cgi?token=exoruikeprgnhct&acc=GSM1262596. Experimentally known target genes of our microRNAs were collected from TarBase 6.0[19]. Enrichment of microRNA targets into pathways was evaluated using the *DIANA-miR Path* web tool available at http://diana.imis.athena-innovation.gr/DianaTools/index.php.

### Incorporation of MVs into CD34^+^ cells

To demonstrate the incorporation of MVs obtained from MSC into human hematopoietic progenitors, CD34^+^ cells obtained by immunomagnetic selection were co-cultured with MVs from MSCs. MVs were labeled with 1μM Vybrant Dil cell-labeling solution (Molecular Probes, life Technology, NY, USA. N° Cat: V22885) during ultracentrifugation at 100,000 g for 70 min at 4°C. After labeling MVs were washed twice under the same conditions in 1X PBS[20, 21] to remove dye excess. MVs were collected, co-cultured with HPC and evaluated at 1, 3, 6, and 24 hours by FC, with the highest rate of incorporation occurring at 24 hours (S2 Fig). Thus, 1X10^5^ CD34^+^ cells were co-cultured for 24 hours with the MSC-derived MVs (30 μg of protein) in a volume of 500μl RPMI per well in all the subsequent experiments to encourage the incorporation (see below).

#### Immunofluorescence

CD34^+^ cells co-cultured with and without MVs were fixed with Carnoy, and nonspecific binding was blocked with 5% of normal donkey serum and bovine serum albumin. To detect MVs incorporated into CD34^+^ cells, a primary antibody rabbit α-CD90 (SC-9163, Santa Cruz Biotechnology, Heidelberg, Germany) was used to identify MSC-derived MVs. Another approach was taken to confirm the incorporation. Thus, MVs from MSC were labeled with Vybrant-Dil cell-labeling solution[20, 21]. As a control experiment, we included an ultracentrifugation tube with only PBS and Vybrant Dil that was processed in the same conditions as the microvesicles and co-cultured with HPC for 24 hours.

Incorporation was evaluated by immunofluorescence, CD34^+^ cells were stained with mouse α-CD45 primary antibody (304002, Biolegend, San Diego, CA). Slides were then incubated for 45min with donkey anti-mouse Alexa Fluor488 and donkey anti-rabbit Alexa Fluor555 (both from Invitrogen, Paisley, UK) and cellular nuclei were stained with DAPI. Slides were mounted using Vectashield H-1000 medium (Vector Laboratories, Inc. Burlingame, CA). For confocal image analysis cells were viewed with a TCS SP5 Confocal Laser Scanning Microscope (Leica Microsystems GMbH, Wetzlar, Germany) with the LAS AF acquisition program (version 2.6.0.7266). In some samples images were acquired with different cell layers (Z-Stacks) of 1μm.

#### MicroRNA expression analysis

To determine whether MSC-derived MVs cargo modifies the microRNAs expression of CD34^+^ cells, miR-10a and miR-15a were analyzed by RT-PCR. Total RNA was extracted from CD34^+^ cells that had been co-cultured with and without MVs (7 MDS and 5 controls) using Trizol reagent (Roche Diagnostics GmbH, Mannheim, Germany) according to the manufacturer’s instructions. We performed individual quantitative PCR for the following microRNAs: Hsa-mir-10a (TM:000387), Hsa-mir-15a (TM:000389) and RNU43 (TM:001095, Applied Biosystems), the latter was used as control. cDNA was prepared to be retrotranscribed with a TaqMan^®^ MicroRNA Reverse Transcription Kit (Applied Biosystems) and the expression was quantified using commercial TaqMan^®^ MicroRNA Expression Assays and the Step One Plus Real-Time PCR System (Applied Biosystems). All samples were performed in duplicate. Relative quantification was calculated from the 2^−ΔCt^ values with the equation: ΔCt = Ct_microRNA_ - Ct_RNU43_. Results were expressed as the ratio between CD34^+^ cells with MVs from MDS or HD and the same CD34^+^ cells without MVs. To evaluate which metabolic pathways are regulated by these two microRNAs DianaLab miR Path web was used.

#### Gene expression analysis

Total RNA was extracted from CD34^+^ cells that had been co-cultured with and without MVs (9 MDS and 6 controls) using Trizol. cDNA was prepared by reverse transcription using the High Capacity kit (Applied Biosystems). Gene expression of *TP53* (Hs01034249-m1), *MDM2* (Hs01066930-m1), and *GADPH* (Hs99999905-m1) as a control gene, was quantified using commercial TaqMan^®^Gene Expression Assays and the Step One Plus Real-Time PCR System (Applied Biosystems). Relative quantification was calculated from the 2^−ΔCt^ values by the equation: ΔCt = Ct_Gene_-Ct_*GADPH*_.

#### Capillary Electrophoresis Immunoassay

Whole cell lysates were obtained from CD34^+^ cells with or without MVs from MDS or healthy donors. Capillary Electrophoresis Immunoassay or Simple Western analyses were performed using the WES™ machine (ProteinSimple Santa Clara, CA) according to the manufacturer’s protocol[22, 23]. In brief, 1.2ng of samples were mixed with a master mix (ProteinSimple) to a final concentration of 1x sample buffer, 1x fluorescent molecular weight markers, and 40mM dithiothreitol (DTT) and then heated at 95°C for 5 min. The samples, blocking reagent, wash buffer, primary antibodies, secondary antibodies, and chemiluminescent substrate were dispensed into designated wells in the manufacturer provided microplate. After plate loading, the separation electrophoresis and immunodetection steps took place in the capillary system and were fully automated. Simple Western analysis was carried out at room temperature, and instrument default settings were used. The data was analyzed with inbuilt Compass software (Proteinsimple), performed normalization of the peak area of protein to peak area of Actin protein in the same sample. For the assay of CD34^+^ cells with or without MVs we used primary antibodies MDM2 (rabbit, polyclonal, thermo scientific #PA5-27209 1:1000) and ACTIN (Mouse, monoclonal, Sigma Aldrich A3854, 1:1000).

#### Cells viability assays

The viability rate was evaluated by FC, after 24hours of co-culture of CD34^+^ cells with MSC-derived MVs (from 10 MDS patients and 10 controls), using APC H7 Annexin V DY634 (Immunostep #ANXVDY, Salamanca, Spain). After 24hours with and without MVs co-cultured cells were harvested, washed and incubated in the Annexin V binding buffer. Annexin V, 7 AAD (# 51-68981E, BD Biosciences) and FITC-conjugated CD34 (11-0349-42, eBioscience, Inc.San Diego, CA) were added, followed by flow cytometric evaluation. Samples were analyzed on a FACSCalibur flow cytometer using Cellquest Pro software (Becton Dickinson). At least 50,000 events/sample were recorded. Data were analyzed using the Infinicyt program (Cytognos). Viable cells were considered if they were not early or late apoptotic cells (APC H7 Annexin V^+^/7AAD^-^ and APC H7 Annexin V^+^/7AAD^+^, respectively).

#### Clonogenic assays

In this experiment 1x10^5^ CD34^+^ cells were co-cultured in a volume of 500μl RPMI per well with or without MVs from 6 HD and 6 patients. After 24hours, 5x10^3^ cells were seeded into methylcellulose MACS Media with Stem cell Factor, GM-CSF, G-CSF, IL-3 and IL-6 (Miltenyi Biotec GmbH, Germany) to quantify the progenitor cell CFU-GM, as previously described[15].

These cultures were incubated in a humidified atmosphere at 37°C with 5% CO_2_. After 14 days, CFU-GM colonies were scored with an inverted microscope. Results were expressed as the ratio between CFU-GM obtained with CD34^+^ cells that had been co-cultured with MVs from MDS or HD and the same CD34^+^ cells without MVs.

### Statistical analysis

Values were summarized as median and range or mean and standard deviation. The Mann-Whitney U-test and Kendall’s test of related samples were used to compare the differences between results. Differences were considered to be significant for values of p<0.05. All statistical analyses were done with SPSS 20.0 (Chicago, IL, USA).

## Results

### MSC, CD34^+^ cells and MVs isolation and characterization

In all the assays, MSC displayed spindle-shaped morphology and fulfilled the minimal criteria for MSC definition required by the ISCT (S3 Fig). The viability of MSC-BM at the time of MVs collection ranged from 86% to 94%.

**Fig 1:**
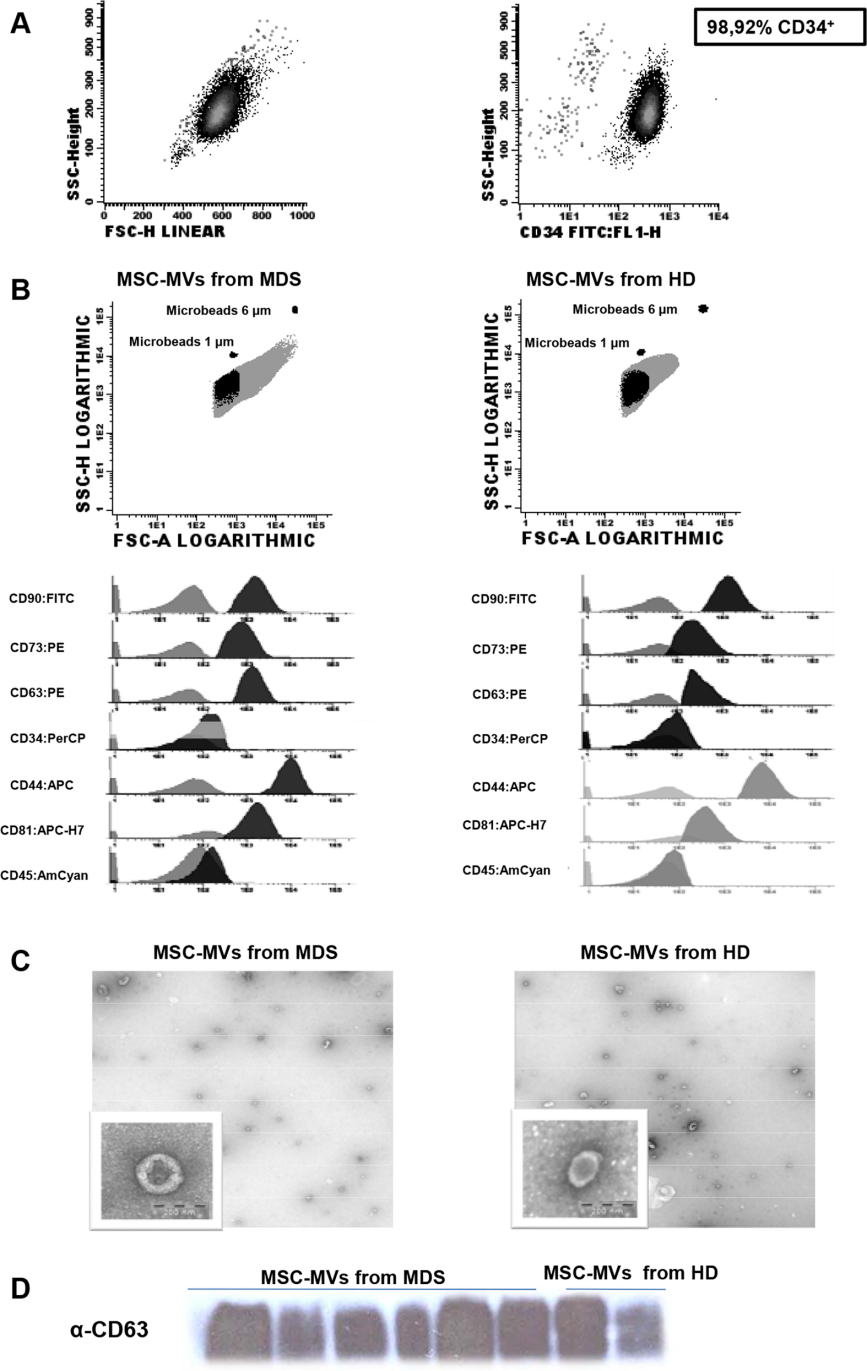
Characterization of CD34^+^ cells from leukapheresis of HD. **(A)** Percentage of CD34^+^ cells isolated by immunomagnetic beads and the purity determined by flow cytometry. **(B)** Flow cytometry characterization of MVs released from MSCs of MDS and HD. The upper images are dot-plots of forward and side scatter of MVs. The gate was defined as elements of smaller size than the 1μm beads. The histograms represent the MVs stained with negative (CD34 and CD45) and positive markers for MVs from HD and MDS-MSC (CD90, CD44, CD73) and for MVs markers (CD81 and CD63). Controls (unstained MVs) are shown in gray; the MVs stained with the different antibodies are shown in black. Images on the left are those of the MVs from MSC-HD, while those on the right images are of the MVs from MSC-MDS. **(C)** Representative images of transmission electronic microscopy of MVs released by MSC from HD(left) and MDS (right) as revealed by TEM. Scale bar, 200nm. Original magnification: x 8000. **(D)** MVs characterization by Western Blot assay for the expression of CD63. HD-MVs: microvesicles from healthy donors. MDS-MVs: microvesicles from patients with myelodysplastic syndrome.

The purity of immunomagnetically selected CD34^+^ cells was over 90% in all cases (Fig 1A) and the mean of the viability of these cells was 90.6%.

Mean size using Nanosight technology was 141.1 for MDS-MVs and 218nm for HD-MVs (S4 Fig). The identification of MVs was similar when Exoquick or ultracentrifugation were performed with the same immunophenotypic profile for MVs isolation and identification. Immunophenotypic analysis by FC showed that all MSC-derived MVs from HD and MDS patients displayed a compared pattern. As it is observed in Fig 1B the logarithmic scale shows that all MVs presented forward scatter intensities less than 1μm beads. They were positively labeled by MSC specific MoAbs (CD90, CD73 and CD44), as well as negative for CD34 and CD45 (Fig 1B). The MVs were positive for exosome markers (CD63 and CD81). MVs without antibody-staining were used to help in establishing the gates to identify the MVs. In order to show that aggregates were not present, S1 Fig shows PBS doubled-filtered staining with the different antibodies, where no positivity was detected in the different channels. In addition, in 6 cases (4 MDS and 2 HD) the presence of MVs in the MSC-derived supernatants was confirmed by transmission electronic microscopy (TEM), whereby these structures were observed to have sizes of around 200nm (Fig 1C). In all cases, Western blot analysis revealed the presence of CD63 in MVs from both patients and controls (Fig 1D).

### MicroRNAs expression is different in MVs from MDS and HD

**Fig 2:**
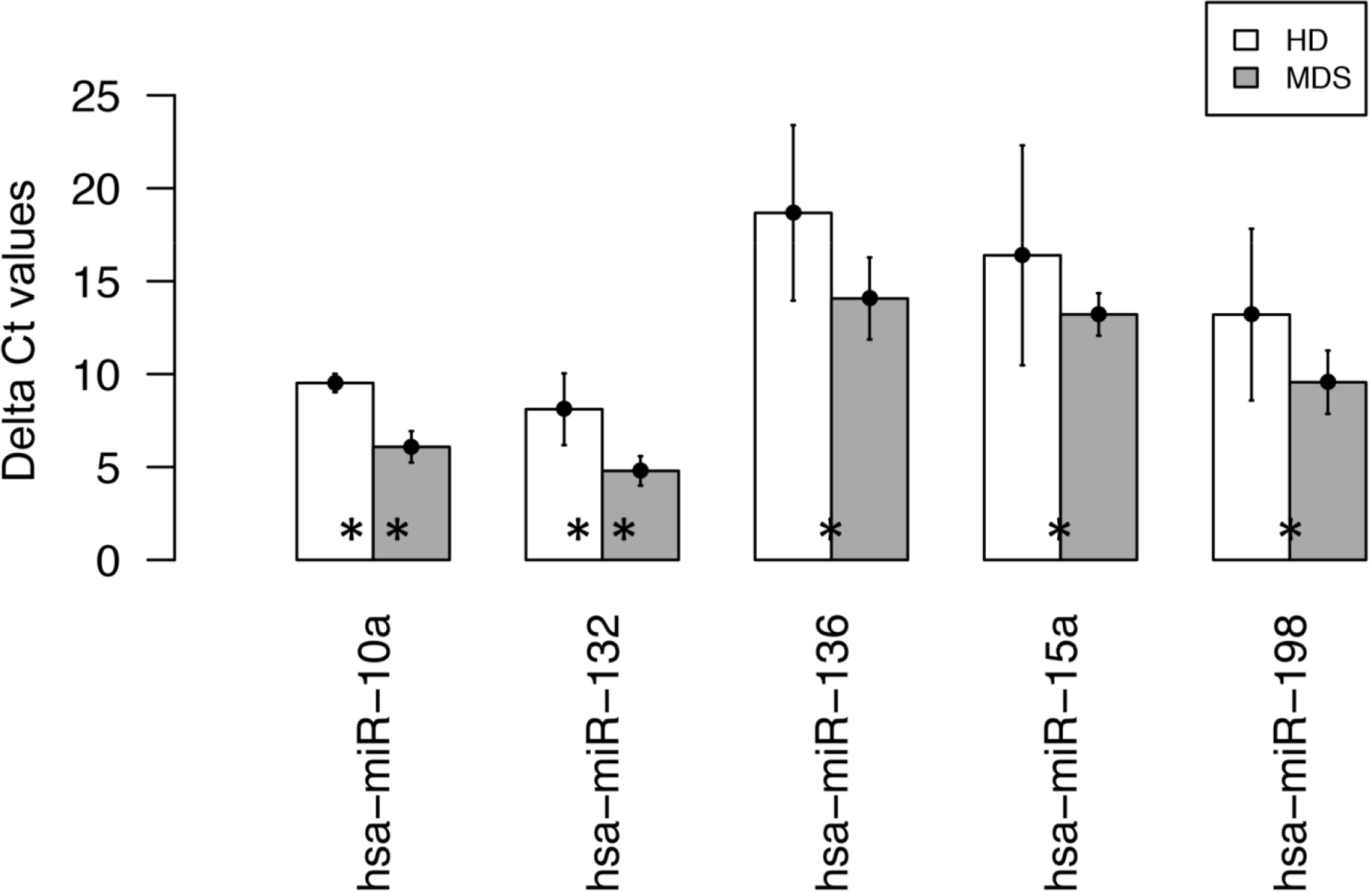
Delta Ct values of 5 microRNAs differentially expressed in MSC-derived MVs between MDS patients and healthy donors (HD). Bars represent median values of Delta Ct per sample category. Mean and confidence interval per sample category are also drawn. Analysis performed over qPCR microRNA arrays. Asterisks denote differential expression p-values: (**) <0.01, (*) <0.05.

To assess whether the microRNA content was different in patients and controls differential expression was analyzed in MSC-MVs from 8 MDS patients and 4 HD on a qPCR *TaqMan* array platform. Statistically significant differences were found in 21 out of 378 tested microRNAs (S5 Fig). Among the 21 significantly expressed microRNAs, 5 of them presented median values within the range of detectable raw Ct levels. Changes in expression based on Delta Ct values of these 5 microRNAs are presented in Fig 2.

In order to ascertain that really some microRNAs were differentially expressed a new normalization using miR-16 as control was performed and 14 microRNAs were overexpressed (S2 Table).

In order to confirm the different expression pattern and as miR-10a and miR-15a were selected because they could be involved in MDS, RT-PCR of both microRNAs was performed. It was verified that microRNA10a expression was significantly higher in MDS-MVs compared to MVs from healthy donor(p≤0.05). MicroRNA 15a overexpression in patients was not statistically significant (although a tendency was observed), may be due to the high variability within the MDS samples (S6 Fig)

### MVs from MSC incorporate into CD34^+^ cells

#### Immunofluorescence

**Fig 3:**
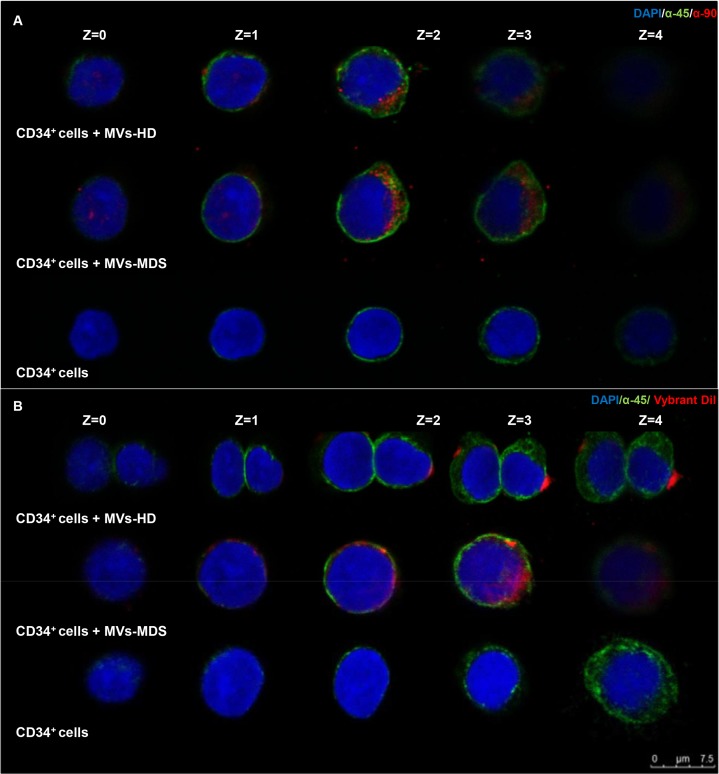
Incorporation of MVs from MSC-MDS and MSC-HD into CD34^+^ cells. **(A)** Representative images of MVs incorporation by CD34^+^ cells stained with anti-CD90 Ab (red) and anti-CD45 Ab (green). **(B)** Representative images of MVs previously labeled with Vybrant-Dil cell-labeling solution (red) that were incorporated into CD34^+^ cells and stained with anti-CD45 Ab (green). **(A-B)** Images in the top row are from CD34^+^ cells that incorporated the MVs released from MSC-HD. Images on the middle row show the incorporation of MVs released from MSC-MDS. In the lower row, images of the CD34^+^ cells (without incorporation) are shown. Nuclei were counterstained with DAPI (blue). Scale bar, 7.5μm. Revealed by confocal microscopy and acquired in layers (z-Stacks) of 1μm.

Once that we have confirmed that some microRNAs were differentially expressed in MVs from patients and controls, we wanted explore whether MVs could be incorporated into hematopoietic progenitors. For this purpose, immunofluorescence was used to evaluate MVs incorporation into CD34^+^ cells obtained from leukapheresis. As shown in Fig 3A, MVs labeled with anti-CD90, an MSC-surface marker (in red), were incorporated into CD34^+^ cells labeled with anti-CD45, a hematopoietic cell marker (in green). When CD34^+^ cells were incubated without MVs, only CD45 expression was observed, while CD90 positivity was absent (Fig 3A).

To confirm these results, experiments using Vybrant-Dil labeled MVs (in red) and CD34^+^ cells with anti-CD45 in green were performed. As it is shown in the Fig 3B, Vybrant-Dil labeled MVs from MSC were incorporated into CD34^+^ cells. The presence of MVs inside the cell was confirmed by capturing several consecutive Z-plane (1μm) pictures.

### Incorporation of MVs into CD34^+^ cells modifies their gene expression

It has been previously published that miR10-a and miR15-a are overexpressed in hematopoietic cells from MDS patients[24]. In Fig 2 we can observe that these two microRNAs are among the most expressed in MVs from our patients and they were selected to assess if their expression was modified in CD34^+^ cells.

**Fig 4:**
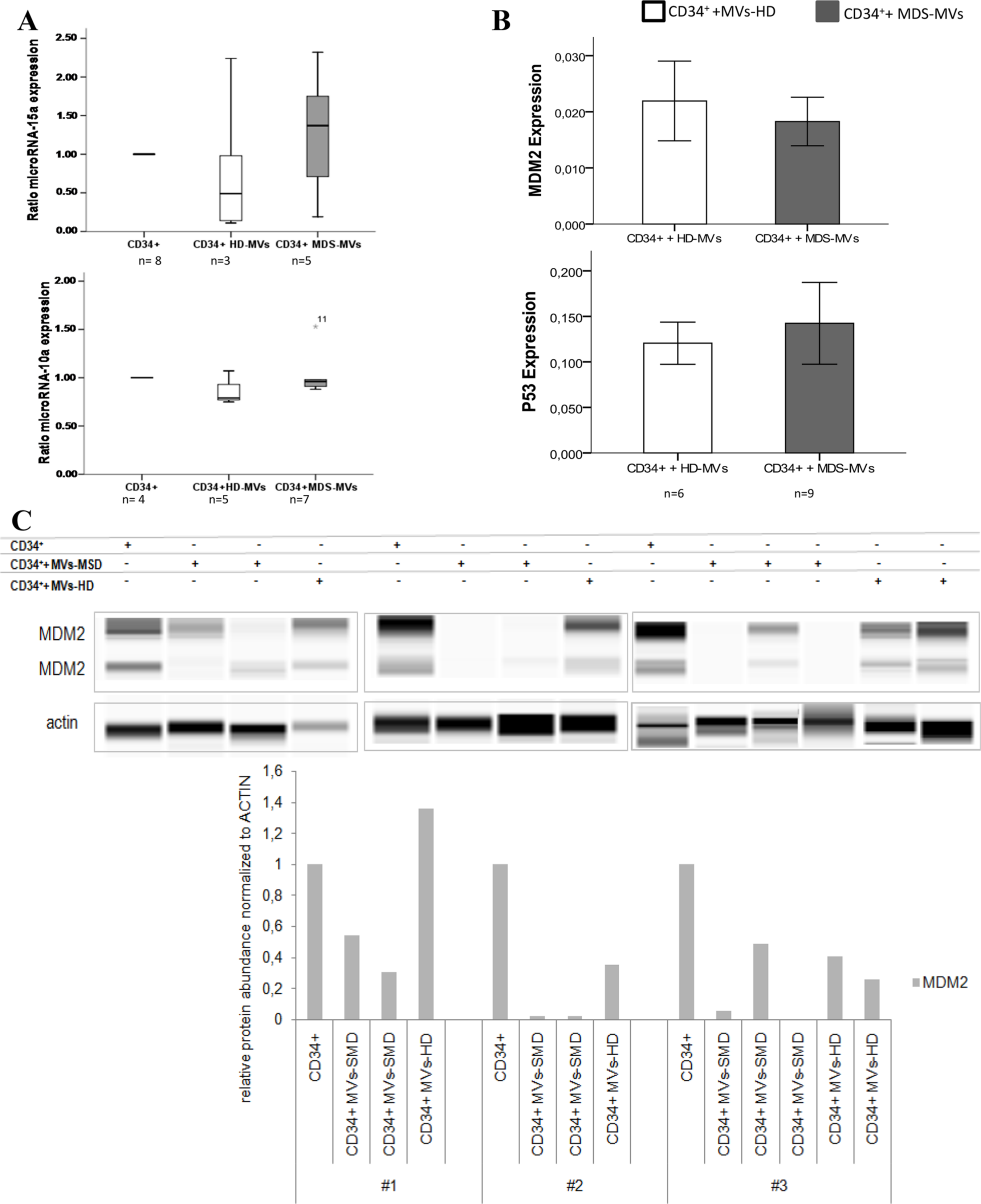
Modification of HPC properties. **(A)** Variations in microRNAs expression when CD34^+^ cells were co-cultured with MDS-MVs or HD-MVs. Ratio was calculated dividing the expression of each microRNA from CD34^+^ + HD-MVs or CD34^+^ + MDS-MVs by that of CD34^+^ cells without MVs. Results were summarized as the median. **(B)** Expression by RT-PCR of *TP53* and *MDM2* in CD34^+^ cells cultured with MDS-MVs from patients (grey) and expression of CD34^+^ cells without MVs (black). Results were summarized as the mean and standard deviation. **(C)** Capillary Electrophoresis Immunoassay of MDM2 vs Actin as control. CD34^+^ cells (without MVs), CD34^+^ cells with MDS-MVs and with HD-MVs. Each bar of the lower graph represents the value of quantified MDM2 protein expression normalized to actin protein abundance. Each bar represents the quantification of both bands of MDM2 from the pseudo-blots, control CD34^+^ cells vs CD34^+^ cells + MDS-MVs or HD-MVs.

The expression of both selected microRNAs–miR-10a and miR-15a- in CD34^+^ cells was analyzed in all cases after incubation with MSC-MVs from MDS patients compared to CD34^+^ cells co-cultured with MVs from normal MSC (Fig 4A). As it is shown in Fig 4A the expression in CD34^+^ cells with MDS-MVs are higher than after the incorporation of HD-MVs.

To analyze which metabolic pathways were regulated by miR-10a and miR-15a, a search in DIANA LAB -Mirpath website (http://diana.imis.athena-innovation.gr/DianaTools/index.php.) was performed. Wnt signaling, cell cycle, MAPK signaling and apoptosis are regulated by these microRNAs. Because it is well known the important role that apoptosis plays into the MDS pathophysiology, this pathway was selected for the subsequent assays.

In order to see whether MSC-derived MVs cargo modifies the expression of some genes in CD34^+^ cells after their incorporation, genes related with apoptosis were selected. For this purpose, *MDM2* and *TP53* were analyzed by RT-PCR (Fig 4B).

The expression of *MDM2* was lower in CD34^+^ cells after incubation with MVs from MDS patients in all experiments, whereas *TP53* expression was higher in this cells when compared with CD34^+^ cells co-cultured with HD-MVs. In order to confirm these features at the protein level the Capillary Electrophoresis Immunoassay was performed. The results are presented as pseudo-blots (Fig 4C) with two bands of MDM2 visible; the upper band corresponds to the full length protein and the lower band corresponds to the cleaved product of MDM2 protein [25]. A significant decrease of MDM2 protein expression in CD34^+^ cells with MVs from MDS was observed (p<0.01) whereas the MDM2 expression was not statistically affected in CD34^+^ cells with HD-MVs (S7 Fig).

Since the content of bioactive molecules was modified into CD34^+^ cells after MVs co-culture, the following step was to study if this incorporation could modify CD34^+^ cells behavior. For this purpose, cell viability and clonogenic potential were studied.

### MVs content also modifies CD34^+^ cells viability as well as CFU-GM production

To assess if the MVs content incorporation could modify HPC behavior, we analyzed cell viability and clonogenic capacity.

For the first purpose, study of viability/apoptosis in CD34^+^ cells with and without incubation with normal or MDS-MVs was performed (n = 10). Compared to the viability of CD34^+^ cells without MVs, incubation with MVs induced an increase in CD34^+^ cells viability in both groups (median increase: 7.9% [range 0.89–19.6] in HD-MVs *vs*.10.2% [range 4.42–24.68] with MDS-MVs). This increase in viability was only statistically significant when cells were incubated with MDS-MVs (p<0.025).

**Fig 5:**
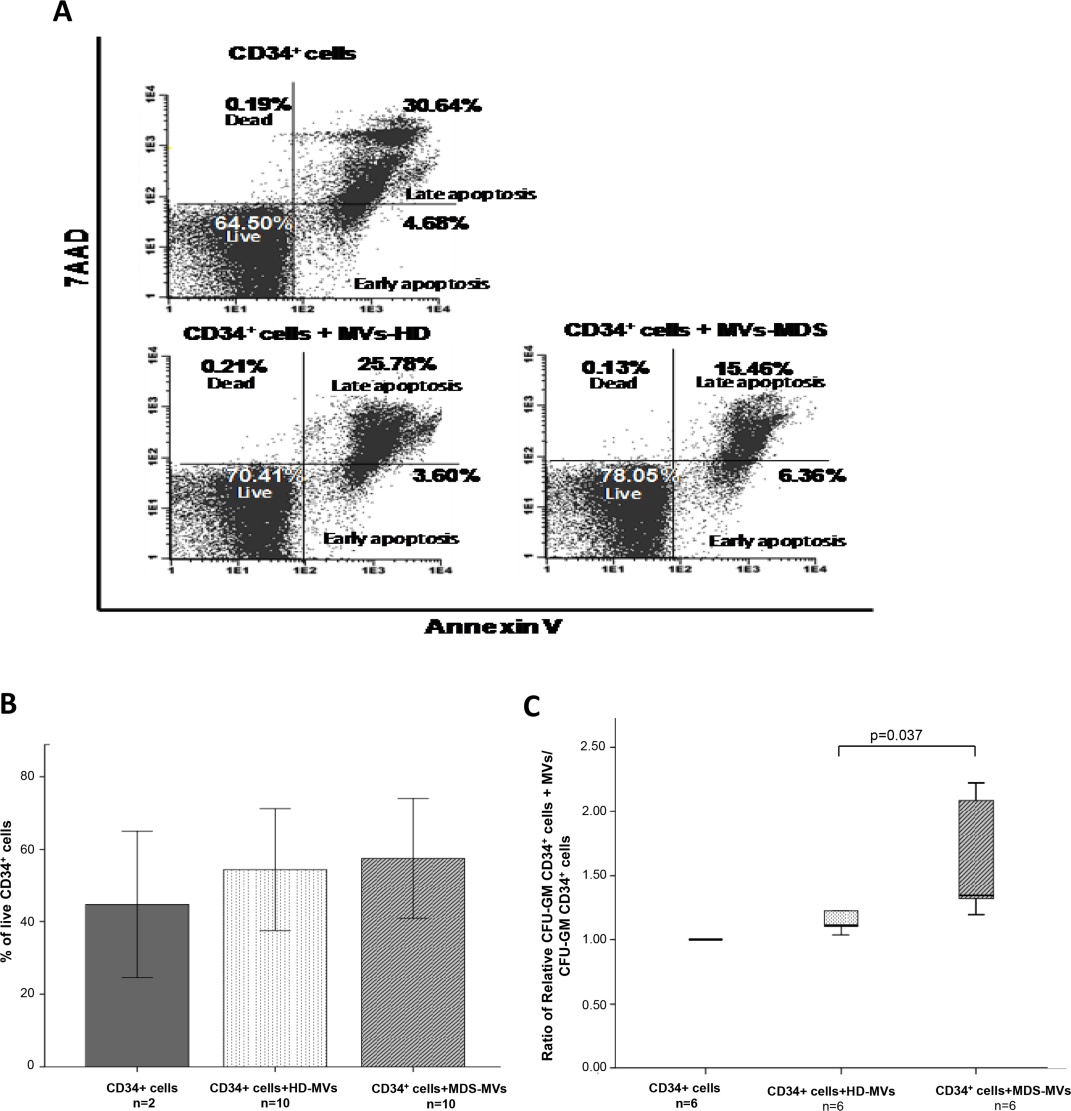
MVs content incorporation results in CD34^+^ cells behavior modification. **(A): Representative FACS plots of annexinV/7AAD staining on CD34**^**+**^ **cells with and without MVs.** Percentage of each subset (dead, live, early and late apoptosis) within the total number of CD34^+^ cells. **(B) Percentage of live CD34**^**+**^ **cells.** An increase on the percentage of CD34^+^ viable cells (annexinV^-^/7AAD^-^) was observed when cells were cultured with MDS-MVs compared with the other groups is shown. **(C) Clonogenic assays.** Results are expressed as the ratio between CFU-GM obtained with CD34^+^ cells that had been cultured with MVs and CD34^+^ cells without MVs.

Regarding CFU-GM production (Fig 5) we observed that the clonogenic capacity of CD34^+^ cells was significantly higher than controls (p = 0.037) when cells had incorporated MDS-MVs (S3 Table). There were no differences in the shape or size of granulo-monocytic colonies between the different experimental groups.

We have not observed any immunophenotypic difference by FACS in CD34^+^ cells versus co-cultured with MDS-MVs or MDS-MVs for any of the markers studied.

## Discussion

The hematopoietic microenvironment is involved in the physiology of the hematopoietic system, but in patients with MDS this microenvironment contributes to the deregulation of hematopoiesis[4]. The mechanisms by which MSC modify HPC from MDS patients are not fully understood. Extracellular vesicles carry cell constituents of the cells of origin, that can be transferred to target cells[12, 26–28]. For example, mRNAs and microRNAs can be transferred to neighboring or distant cells via fusion of the exosome to the target cell membrane[29]. MVs have therefore been described as a novel mechanism of cell-to-cell communication.

Other researchers have demonstrated that BM-MSC can release MVs which are transferred to other cells, thereby modifying them[30]. It has recently been reported that MVs from BM-MSC may be involved in multiple myeloma progression and drug resistance[31, 32].

In the present work we hypothesized that microvesicles produced by BM-MSC from MDS patients are involved in the relationship between the BM microenvironment and hematopoietic cells, thereby contributing to the intercellular communication.

In order to avoid the great variability that is characteristic of MDS patients, only low-risk patients were included.

First, we tried to obtain and characterize MVs from MSC from MDS patients and HDs which were expanded and stressed by serum deprivation to provide sufficient MVs to perform the various studies. Then they were obtained from culture supernatants by two approaches: Exoquick-TC™, a commercial assay, and ultracentrifugation. The identification of MVs after obtaining them by these two approaches showed similar results. In order to reduce background, the majority of experiments were performed by ultracentrifugation, but, when a high quantity of mRNA was needed, Exoquick assay was used.

MVs were characterized by FC. In order to differentiate true MVs events from background noise we defined MVs as particles that were less than 1μm and were also positive for MSC-specific immunophenotypic markers. More details of MVs identification by flow cytometry are provided in other paper that has been recently submitted[33].

We included in the panel of MoAbs one marker that is always expressed by exosomes, such as CD63 or CD81[17]. Since MVs express some surface antigens on the membrane of their cell of origin, the other selected antibodies were against typical MSC-positive surface markers. CD45 and CD34 were included as negative markers. This panel enabled the identification of these structures in all cases and when they were obtained by ultracentrifugation and Exoquick-TC™.

TEM confirmed that these structures were MVs. Fig 1B shows that these structures were present in all tested cases and had similar features to those noted by other groups[12, 34].

It would be interesting to analyze if different MDS subtypes show differences in their MVs. However, due to the great variability in these diseases, a very high number of patients should be included and this approach exceeds the aim of the present work.

The confocal microscopy assay suggests that HD-MVs and MDS-MVs are both able to become incorporated into CD34^+^ cells. The Z-Stack imaging results also support this notion.

As previously stated, MSC-derived MVs can incorporate into neighbor cells and modify their behavior by transferring microRNAs, mRNA and proteins. MicroRNAs are small non-coding RNAs involved in the regulation of gene expression and have a crucial role in the regulation of hematopoiesis[35]. MicroRNAs can play a role in the development of some malignant hematopoietic disorders such as MDS[24]. Evidence that microRNAs deregulation in the microenvironment is involved in MDS pathogenesis comes from the seminal study of Raaijmaikers et al[9], who demonstrated that, when *DICER-1* was deleted in murine osteoprogenitors, these animals developed an MDS similar to the human disease. Subsequently, we reported that the level of expression of *DICER-1* was lower in MSC from MDS patients, altering the microRNA content in MSCs from MDS patients compared with MSC from HD[10]. Because CD34^+^ cells is a heterogeneous cell population. The analysis of the effect of MSC-MVs into different cell subsets could be interesting. However, the low number of CD34^+^ cells into the BM makes very difficult to sort all the CD34^+^ cells subtypes and is out of the scope of this study. New approaches in order to respond to these questions are warranted.

It has also been clearly demonstrated that microRNAs are involved in the pathogenesis of MDS[24, 36]. In this context, it could be hypothesized that the microRNAs cargo in MVs may differ between MDS-MSC and HD-MSC, and could be transferred into hematopoietic progenitors. To test this hypothesis, microRNA-expression arrays were performed in MSC-derived MVs from MDS patients and HD. A significantly different content was observed, with 21 microRNAs in MVs from patients more strongly expressed. In order to confirm the different expression pattern of microRNAs in MVs from patients and donors different normalization methods have been used. The increased expression of microRNAs is a rather unexpected feature since MSC from MDS patients have an overall lower level of microRNAs expression compared with those from HD, as we have previously demonstrated[10], implying that the microRNAs cargo in MVs does not arise as a random event but from a selective mechanism that probably exists for intercellular communication. This selective process has been previously shown for these structures[12]. MicroR-10a and miR-15a are two of the most overexpressed microRNAs in the MSC-derived MVs from MDS patients. So, we wanted to establish whether the incorporation of MVs modified microRNA expression cell. We were able to demonstrate that CD34^+^ cells indeed showed increased expression of microRNA-10a and a tendency to increased expression of microRNA-15a when they had been in contact with MSC-MVs from MDS patients compared with HD. Why HD showed a decreased expression when compared with CD34+ cells without MVs is difficult to explain since MVs are carrying many bioactive molecules that could have different effects in the recipient cell. MicroR-10a and miR-15a are both known to be overexpressed in hematopoietic cells of MDS patients[37, 38]. This microRNAs overexpression could be due, at least in part, to their transfer from MSC to HPC by MVs. In this context, our findings about HPC overexpression when they had been in contact with these structures point to this mechanism. Given that microRNAs are involved in gene expression regulation we tried to establish which genes and pathways are regulated by these two microRNAs. Cell cycle, cancer, TP53 and PI3K/AKT were among those identified. *TP53* is involved in very important cell functions in the hematopoietic system and is constantly regulated in cells[39]. *MDM2* is a very important regulator of *TP53* in the hematopoietic system and it has been shown that this gene is necessary to rescue erythroid progenitors from *TP53* mediated apoptosis[40] as well as to control ROS induced TP53 levels in the hematopoietic system[41]. To see whether MDM2 could be down-regulated in CD34^+^ cells that had been in contact with miR10a and miR15a from MVs, we examined whether their increased levels of gave rise to a modification of *MDM2* gene expression. *MDM2* was decreased in CD34^+^ cells when MDS-MVs content was incorporated. Concomitantly, *TP53* was increased, suggesting that, at least in some cases, the increased erythroid progenitor apoptosis seen in MDS could be mediated by MVs from the microenvironment carrying microRNAs acting on the TP53 pathway.

Over the last years, considerable information about the role of MVs in intercellular communication has been published showing that these structures are involved in both, physiological and pathological processes[42]. Also information about the role of microenvironment in the pathophysiology of hematopoietic neoplasms have been published by our group [5, 33, 43] and other teams [43–45].

More specifically, it has been shown in other cancer models based on similar approaches that MVs are involved in cancer cell protection and disease progression[46] through the provision of a favorable microenvironment. In the present work we have shown that this mechanism could be involved. MVs from MSC seem to be delivered into the microenvironment ant their content incorporated into HPC. Among the incorporated bioactive molecules there are microRNAs such as miR10a and miR15a involved in very important cell functions: cell cycle proliferation, apoptosis, etc. and we have confirmed that they could modify some hematopoietic cell properties. In fact, we found that MVs from MDS patients increased not only CD34^+^ cells viability but also their clonogenic capacity relative to the same CD34^+^ cells with MVs from HD. These results suggest that these structures could also act as a survival mechanism for MDS clonal CD34^+^ progenitor cells.

In summary, our results show that BM-MSC from MDS release MVs that incorporate into HPC, delivering bioactive molecules that could modify their genetic expression pattern and increase their viability and clonogenicity. These MVs could be involved in the maintenance of clonal hematopoiesis in MDS patients. These experiments show, once again, that the microenvironment has an important role in maintaining neoplastic diseases.

## Supporting Information

S1 Fig Flow cytometry characterization of MVs released from MSC of MDS and HD A: Dot-Plots MDS-MVs; B: Dot-Plots HD-MVs; C: Dot-Plots of double filtered PBS. (TIF)

S2 Fig Representative dot plots of the sequence of MVs (MDS-MVs and HD-MVs) incorporation into CD34^+^ cells. The consecutive images represent the CD34^+^ cells that were incubated with MVs labeled with Vybrant Dil cell-labeling solution and evaluated at 1, 3, 6, and 24 hours by FC.
(TIF)

S3 Fig MSC characterization. Adipogenic (left) and osteogenic (right) differentiation of MSC from patients with myelodysplastic syndromes. B) Flow cytometry characterization of MSCs from MDS.
(TIF)

S4 Fig Nanosight analysis of one sample. (TIF)

S5 Fig Heatmap based on Delta Ct values of 21 microRNAs increased in MSC-MVs from MDS patients. Upper, a dendrogram of sample-to-sample Euclidean distances. At the side, a dendrogram of microRNA Euclidean distances. HD, healthy donors; MDS, myelodysplastic syndromes.
(TIF)

S6 Fig MicroRNA expression by RT-PCR of miR-10a and miR-15a between MDS-MVs and HD-MVs. Results expressed as median.
(TIF)

S7 Fig MDM2 protein expression analysis in CD34^+^ cells (without MV), CD34^+^ cells with MV from MDS (CD34^+^+MVs-MDS) and with MV from HD (CD34^+^+MVs-HD). **p<0.01 as assessed by t-test student.
(TIF)

S1 Methods (DOCX)

S1 Table Patients included into all studies. (DOCX)

S2 Table *14 microRNAs differentially expressed*. (DOCX)

S3 Table Number of CFU-GM/5000 CD34^+^ cells. (DOCX)

## References

[1] Corey SJ, Minden MD, Barber DL, Kantarjian H, Wang JC, Schimmer AD. Myelodysplastic syndromes: the complexity of stem-cell diseases. Nature reviews Cancer. 2007;7(2):118–29. Epub 2007/01/26. 10.1038/nrc2047 . 17251918

[2] Nimer SD. Myelodysplastic syndromes. Blood. 2008;111(10):4841–51. Epub 2008/05/10. 10.1182/blood-2007-08-078139 . 18467609

[3] Issa JP. The myelodysplastic syndrome as a prototypical epigenetic disease. Blood. 2013;121(19):3811–7. Epub 2013/05/11. 10.1182/blood-2013-02-451757 23660859 PMC3650703

[4] Tauro S, Hepburn MD, Bowen DT, Pippard MJ. Assessment of stromal function, and its potential contribution to deregulation of hematopoiesis in the myelodysplastic syndromes. Haematologica. 2001;86(10):1038–45. Epub 2001/10/17. . 11602409

[5] Lopez-Villar O, Garcia JL, Sanchez-Guijo FM, Robledo C, Villaron EM, Hernandez-Campo P, et al Both expanded and uncultured mesenchymal stem cells from MDS patients are genomically abnormal, showing a specific genetic profile for the 5q- syndrome. Leukemia. 2009;23(4):664–72. Epub 2009/01/20. 10.1038/leu.2008.361 . 19151777

[6] Blau O, Baldus CD, Hofmann WK, Thiel G, Nolte F, Burmeister T, et al Mesenchymal stromal cells of myelodysplastic syndrome and acute myeloid leukemia patients have distinct genetic abnormalities compared with leukemic blasts. Blood. 2011;118(20):5583–92. Epub 2011/09/29. 10.1182/blood-2011-03-343467 21948175 PMC3217359

[7] Flores-Figueroa E, Montesinos JJ, Flores-Guzman P, Gutierrez-Espindola G, Arana-Trejo RM, Castillo-Medina S, et al Functional analysis of myelodysplastic syndromes-derived mesenchymal stem cells. Leukemia research. 2008;32(9):1407–16. Epub 2008/04/15. 10.1016/j.leukres.2008.02.013 . 18405968

[8] Zhao ZG, Xu W, Yu HP, Fang BL, Wu SH, Li F, et al Functional characteristics of mesenchymal stem cells derived from bone marrow of patients with myelodysplastic syndromes. Cancer letters. 2012;317(2):136–43. Epub 2012/01/14. 10.1016/j.canlet.2011.08.030 . 22240014

[9] Raaijmakers MH, Mukherjee S, Guo S, Zhang S, Kobayashi T, Schoonmaker JA, et al Bone progenitor dysfunction induces myelodysplasia and secondary leukaemia. Nature. 2010;464(7290):852–7. Epub 2010/03/23. 10.1038/nature08851 20305640 PMC3422863

[10] Santamaria C, Muntion S, Roson B, Blanco B, Lopez-Villar O, Carrancio S, et al Impaired expression of DICER, DROSHA, SBDS and some microRNAs in mesenchymal stromal cells from myelodysplastic syndrome patients. Haematologica. 2012;97(8):1218–24. Epub 2012/03/01. 10.3324/haematol.2011.054437 22371183 PMC3409820

[11] Yuana Y, Sturk A, Nieuwland R. Extracellular vesicles in physiological and pathological conditions. Blood reviews. 2013;27(1):31–9. Epub 2012/12/25. 10.1016/j.blre.2012.12.002 . 23261067

[12] Collino F, Deregibus MC, Bruno S, Sterpone L, Aghemo G, Viltono L, et al Microvesicles derived from adult human bone marrow and tissue specific mesenchymal stem cells shuttle selected pattern of miRNAs. PloS one. 2010;5(7):e11803 Epub 2010/07/30. 10.1371/journal.pone.0011803 20668554 PMC2910725

[13] Montecalvo A, Larregina AT, Shufesky WJ, Stolz DB, Sullivan ML, Karlsson JM, et al Mechanism of transfer of functional microRNAs between mouse dendritic cells via exosomes. Blood. 2012;119(3):756–66. Epub 2011/10/28. 10.1182/blood-2011-02-338004 22031862 PMC3265200

[14] Dominici M, Le Blanc K, Mueller I, Slaper-Cortenbach I, Marini F, Krause D, et al Minimal criteria for defining multipotent mesenchymal stromal cells. The International Society for Cellular Therapy position statement. Cytotherapy. 2006;8(4):315–7. Epub 2006/08/23. 10.1080/14653240600855905 . 16923606

[15] Villaron EM, Almeida J, Lopez-Holgado N, Sanchez-Guijo FM, Alberca M, Blanco B, et al In leukapheresis products from non-Hodgkin's lymphoma patients, the immature hematopoietic progenitors show higher CD90 and CD34 antigenic expression. Transfusion and apheresis science: official journal of the World Apheresis Association: official journal of the European Society for Haemapheresis. 2007;37(2):145–56. Epub 2007/11/07. 10.1016/j.transci.2007.05.001 . 17983836

[16] Thery C, Amigorena S, Raposo G, Clayton A. Isolation and characterization of exosomes from cell culture supernatants and biological fluids. Current protocols in cell biology / editorial board, Juan S Bonifacino [et al]. 2006;Chapter 3:Unit 3 22. Epub 2008/01/30. 10.1002/0471143030.cb0322s30 . 18228490

[17] Gelderman MP, Simak J. Flow cytometric analysis of cell membrane microparticles. Methods Mol Biol. 2008;484:79–93. Epub 2008/07/02. 10.1007/978-1-59745-398-1_6 . 18592174

[18] Dvinge H, Bertone P. HTqPCR: high-throughput analysis and visualization of quantitative real-time PCR data in R. Bioinformatics. 2009;25(24):3325–6. Epub 2009/10/08. 10.1093/bioinformatics/btp578 19808880 PMC2788924

[19] Vergoulis T, Vlachos IS, Alexiou P, Georgakilas G, Maragkakis M, Reczko M, et al TarBase 6.0: capturing the exponential growth of miRNA targets with experimental support. Nucleic acids research. 2012;40(Database issue):D222–9. Epub 2011/12/03. 10.1093/nar/gkr1161 22135297 PMC3245116

[20] Grange C, Tapparo M, Bruno S, Chatterjee D, Quesenberry PJ, Tetta C, et al Biodistribution of mesenchymal stem cell-derived extracellular vesicles in a model of acute kidney injury monitored by optical imaging. International journal of molecular medicine. 2014;33(5):1055–63. Epub 2014/02/28. 10.3892/ijmm.2014.1663 24573178 PMC4020482

[21] Herrera Sanchez MB, Bruno S, Grange C, Tapparo M, Cantaluppi V, Tetta C, et al Human liver stem cells and derived extracellular vesicles improve recovery in a murine model of acute kidney injury. Stem cell research & therapy. 2014;5(6):124 Epub 2014/11/12. 10.1186/scrt514 . 25384729 PMC4446072

[22] Gentalen ET, Proctor JM. Using the Peggy Simple Western system for fine needle aspirate analysis. Methods Mol Biol. 2015;1219:139–55. Epub 2014/10/14. 10.1007/978-1-4939-1661-0_11 . 25308267

[23] Rustandi RR, Anderson C, Hamm M. Application of capillary electrophoresis in glycoprotein analysis. Methods Mol Biol. 2013;988:181–97. Epub 2013/03/12. 10.1007/978-1-62703-327-5_11 . 23475720

[24] Fang J, Varney M, Starczynowski DT. Implication of microRNAs in the pathogenesis of MDS. Current pharmaceutical design. 2012;18(22):3170–9. Epub 2012/05/11. . 22571695 10.2174/1381612811209023170 PMC4863958

[25] Chen L, Marechal V, Moreau J, Levine AJ, Chen J. Proteolytic cleavage of the mdm2 oncoprotein during apoptosis. The Journal of biological chemistry. 1997;272(36):22966–73. Epub 1997/09/05. . 9278461 10.1074/jbc.272.36.22966

[26] Valadi H, Ekstrom K, Bossios A, Sjostrand M, Lee JJ, Lotvall JO. Exosome-mediated transfer of mRNAs and microRNAs is a novel mechanism of genetic exchange between cells. Nature cell biology. 2007;9(6):654–9. Epub 2007/05/09. 10.1038/ncb1596 . 17486113

[27] Yuan A, Farber EL, Rapoport AL, Tejada D, Deniskin R, Akhmedov NB, et al Transfer of microRNAs by embryonic stem cell microvesicles. PloS one. 2009;4(3):e4722 Epub 2009/03/07. 10.1371/journal.pone.0004722 19266099 PMC2648987

[28] Gajos-Michniewicz A, Duechler M, Czyz M. MiRNA in melanoma-derived exosomes. Cancer letters. 2014;347(1):29–37. Epub 2014/02/12. 10.1016/j.canlet.2014.02.004 . 24513178

[29] Hannafon BN, Ding WQ. Intercellular Communication by Exosome-Derived microRNAs in Cancer. International journal of molecular sciences. 2013;14(7):14240–69. Epub 2013/07/11. 10.3390/ijms140714240 23839094 PMC3742242

[30] Bruno S, Collino F, Deregibus MC, Grange C, Tetta C, Camussi G. Microvesicles derived from human bone marrow mesenchymal stem cells inhibit tumor growth. Stem cells and development. 2013;22(5):758–71. Epub 2012/10/05. 10.1089/scd.2012.0304 . 23034046

[31] Roccaro AM, Sacco A, Maiso P, Azab AK, Tai YT, Reagan M, et al BM mesenchymal stromal cell-derived exosomes facilitate multiple myeloma progression. The Journal of clinical investigation. 2013;123(4):1542–55. Epub 2013/03/05. 10.1172/JCI66517 23454749 PMC3613927

[32] Wang J, Hendrix A, Hernot S, Lemaire M, De Bruyne E, Van Valckenborgh E, et al Bone marrow stromal cell-derived exosomes as communicators in drug resistance in multiple myeloma cells. Blood. 2014;124(4):555–66. Epub 2014/06/15. 10.1182/blood-2014-03-562439 . 24928860

[33] Ramos TL, Sánchez-Abarca LI, Muntión S, Preciado S, Puig Morón N, López-Ruano G, et al Human mesenchymal stromal cell (hMSC)-derived extracellular vesicles: Optimization of immunophenotypic characterization. Cell Communications and Signalling. 2015.

[34] Aliotta JM, Sanchez-Guijo FM, Dooner GJ, Johnson KW, Dooner MS, Greer KA, et al Alteration of marrow cell gene expression, protein production, and engraftment into lung by lung-derived microvesicles: a novel mechanism for phenotype modulation. Stem Cells. 2007;25(9):2245–56. Epub 2007/06/09. 10.1634/stemcells.2007-0128 17556595 PMC3376082

[35] Havelange V, Garzon R. MicroRNAs: emerging key regulators of hematopoiesis. American journal of hematology. 2010;85(12):935–42. Epub 2010/10/14. 10.1002/ajh.21863 . 20941782

[36] Vasilatou D, Papageorgiou SG, Dimitriadis G, Pappa V. Epigenetic alterations and microRNAs: new players in the pathogenesis of myelodysplastic syndromes. Epigenetics: official journal of the DNA Methylation Society. 2013;8(6):561–70. Epub 2013/06/14. 10.4161/epi.24897 23760524 PMC3857336

[37] Li X, Xu F, Chang C, Byon J, Papayannopoulou T, Deeg HJ, et al Transcriptional regulation of miR-10a/b by TWIST-1 in myelodysplastic syndromes. Haematologica. 2013;98(3):414–9. Epub 2012/09/18. 10.3324/haematol.2012.071753 22983574 PMC3659943

[38] Pons A, Nomdedeu B, Navarro A, Gaya A, Gel B, Diaz T, et al Hematopoiesis-related microRNA expression in myelodysplastic syndromes. Leukemia & lymphoma. 2009;50(11):1854–9. Epub 2009/11/04. 10.3109/10428190903147645 . 19883312

[39] Abbas HA, Pant V, Lozano G. The ups and downs of p53 regulation in hematopoietic stem cells. Cell Cycle. 2011;10(19):3257–62. Epub 2011/10/01. 10.4161/cc.10.19.17721 21957490 PMC3233622

[40] Maetens M, Doumont G, Clercq SD, Francoz S, Froment P, Bellefroid E, et al Distinct roles of Mdm2 and Mdm4 in red cell production. Blood. 2007;109(6):2630–3. Epub 2006/11/16. 10.1182/blood-2006-03-013656 . 17105817

[41] Abbas HA, Maccio DR, Coskun S, Jackson JG, Hazen AL, Sills TM, et al Mdm2 is required for survival of hematopoietic stem cells/progenitors via dampening of ROS-induced p53 activity. Cell stem cell. 2010;7(5):606–17. Epub 2010/11/03. 10.1016/j.stem.2010.09.013 21040902 PMC3026610

[42] Corrado C, Raimondo S, Saieva L, Flugy AM, De Leo G, Alessandro R. Exosome-mediated crosstalk between chronic myelogenous leukemia cells and human bone marrow stromal cells triggers an interleukin 8-dependent survival of leukemia cells. Cancer letters. 2014;348(1–2):71–6. Epub 2014/03/25. 10.1016/j.canlet.2014.03.009 . 24657661

[43] Pereira JK, Traina F, Machado-Neto JA, Duarte Ada S, Lopes MR, Saad ST, et al Distinct expression profiles of MSI2 and NUMB genes in myelodysplastic syndromes and acute myeloid leukemia patients. Leukemia research. 2012;36(10):1300–3. Epub 2012/07/13. 10.1016/j.leukres.2012.06.010 . 22784712

[44] Schepers K, Pietras EM, Reynaud D, Flach J, Binnewies M, Garg T, et al Myeloproliferative neoplasia remodels the endosteal bone marrow niche into a self-reinforcing leukemic niche. Cell stem cell. 2013;13(3):285–99. Epub 2013/07/16. 10.1016/j.stem.2013.06.009 23850243 PMC3769504

[45] Geyh S, Oz S, Cadeddu RP, Frobel J, Bruckner B, Kundgen A, et al Insufficient stromal support in MDS results from molecular and functional deficits of mesenchymal stromal cells. Leukemia. 2013;27(9):1841–51. Epub 2013/06/26. 10.1038/leu.2013.193 . 23797473

[46] Muralidharan-Chari V, Clancy JW, Sedgwick A, D'Souza-Schorey C. Microvesicles: mediators of extracellular communication during cancer progression. Journal of cell science. 2010;123(Pt 10):1603–11. Epub 2010/05/07. 10.1242/jcs.064386 20445011 PMC2864708

