# Supplementary figures and images for: Microvesicles from Mesenchymal Stromal Cells Are Involved in HPC-Microenvironment Crosstalk in Myelodysplastic Patients
Source: PLoS One. 2016 Feb 2;11(2):e0146722. doi: 10.1371/journal.pone.0146722 (PMC4737489; doi:10.1371/journal.pone.0146722)

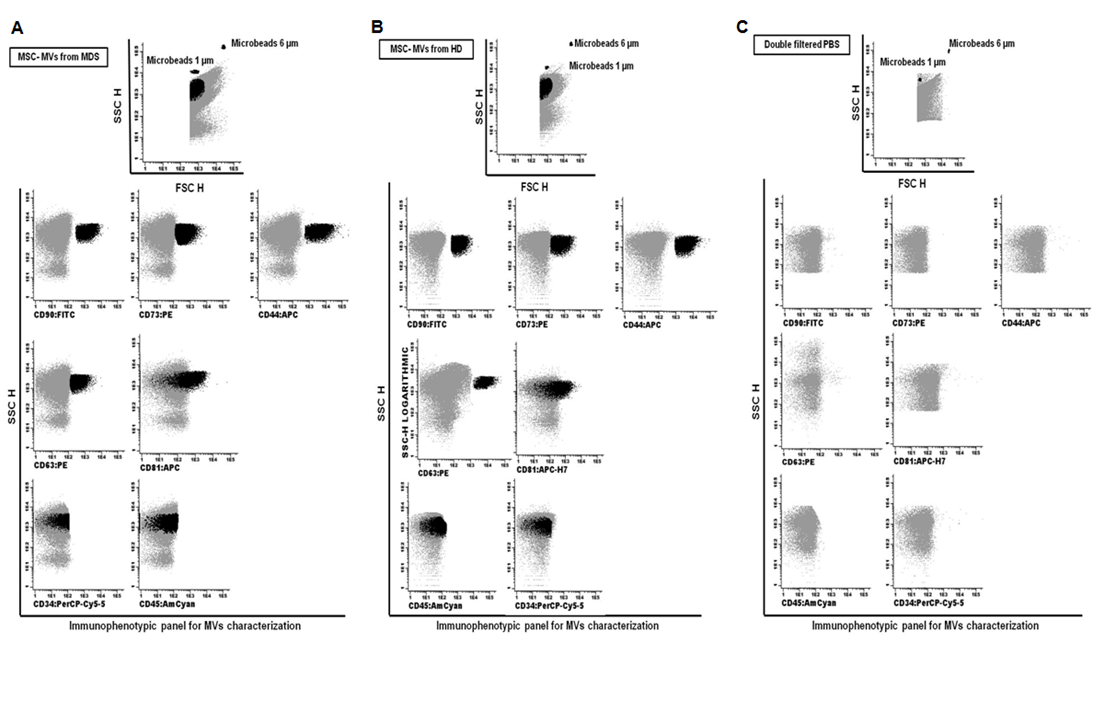

Supplement: S1 Fig — (TIF) [file pone.0146722.s001.tif]

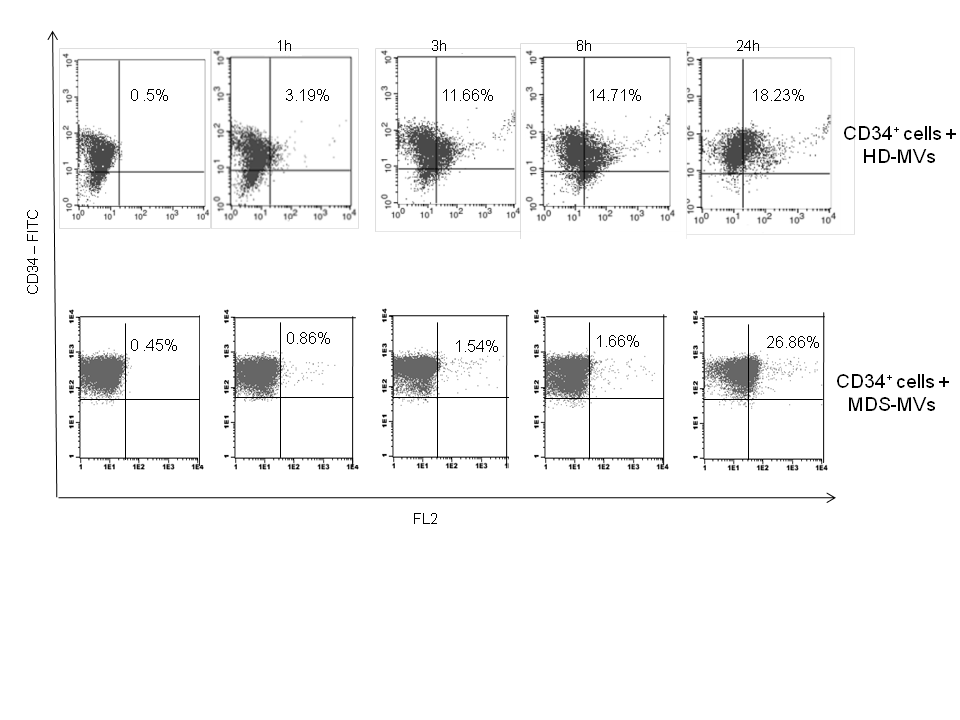

Supplement: S2 Fig — The consecutive images represent the CD34+ cells that were incubated with MVs labeled with Vybrant Dil cell-labeling solution and evaluated at 1, 3, 6, and 24 hours by FC. (TIF) [file pone.0146722.s002.tif]

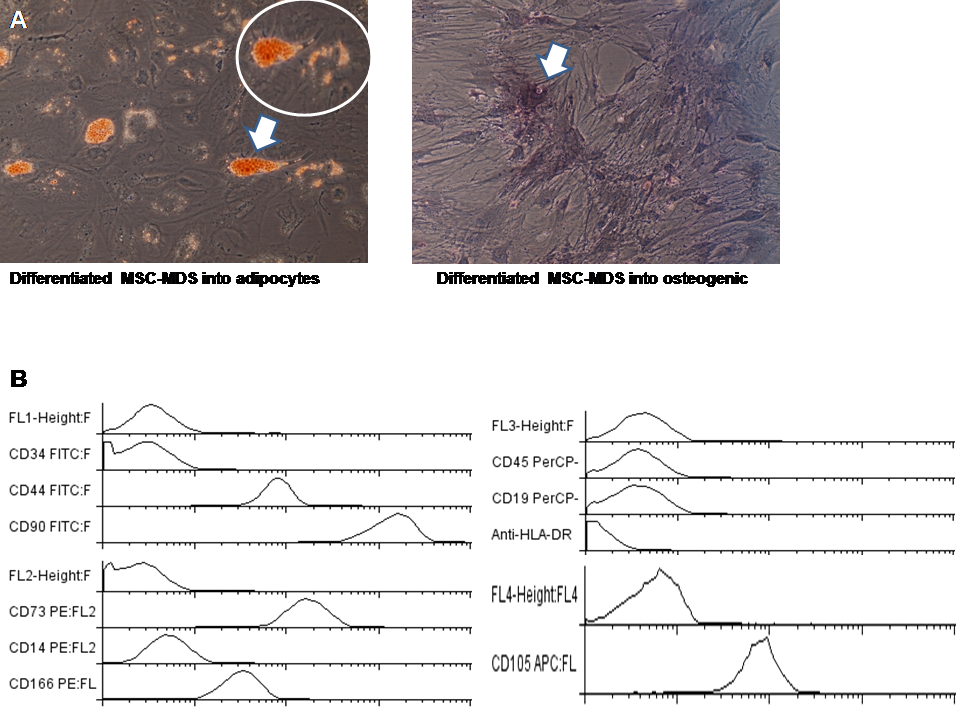

Supplement: S3 Fig — Adipogenic (left) and osteogenic (right) differentiation of MSC from patients with myelodysplastic syndromes. B) Flow cytometry characterization of MSCs from MDS. (TIF) [file pone.0146722.s003.tif]

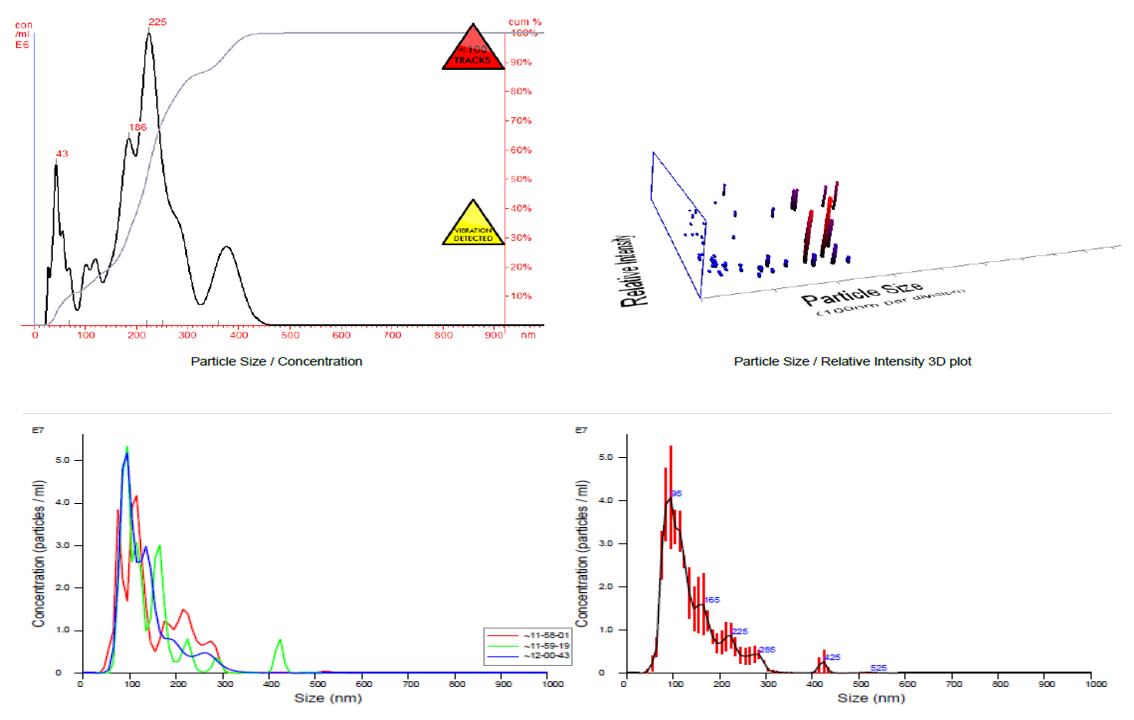

Supplement: S4 Fig — (TIF) [file pone.0146722.s004.tif]

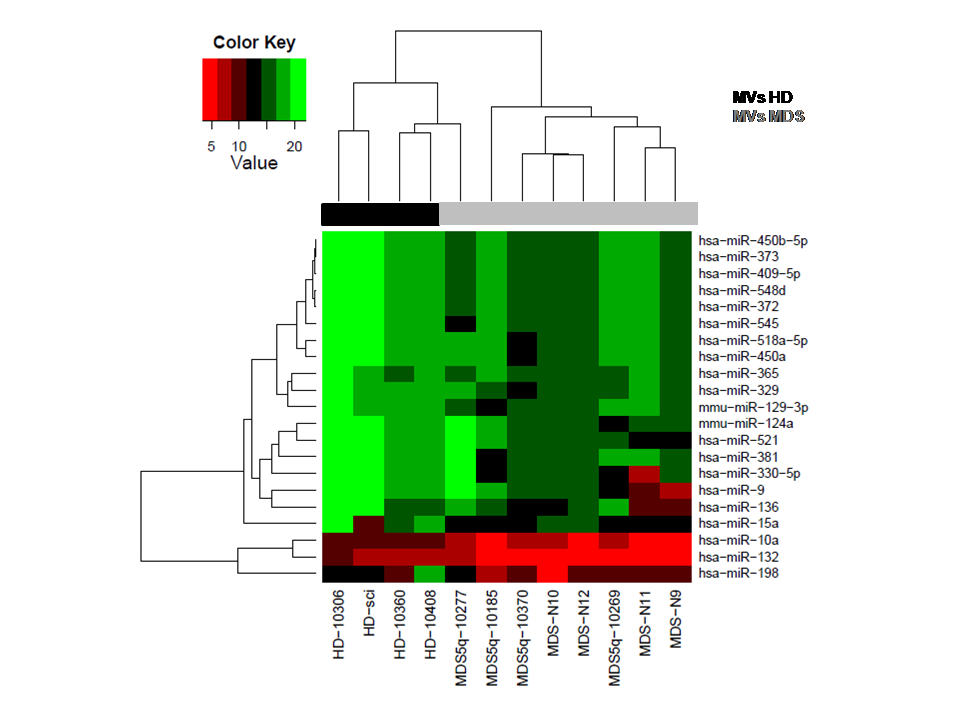

Supplement: S5 Fig — Upper, a dendrogram of sample-to-sample Euclidean distances. At the side, a dendrogram of microRNA Euclidean distances. HD, healthy donors; MDS, myelodysplastic syndromes. (TIF) [file pone.0146722.s005.tif]

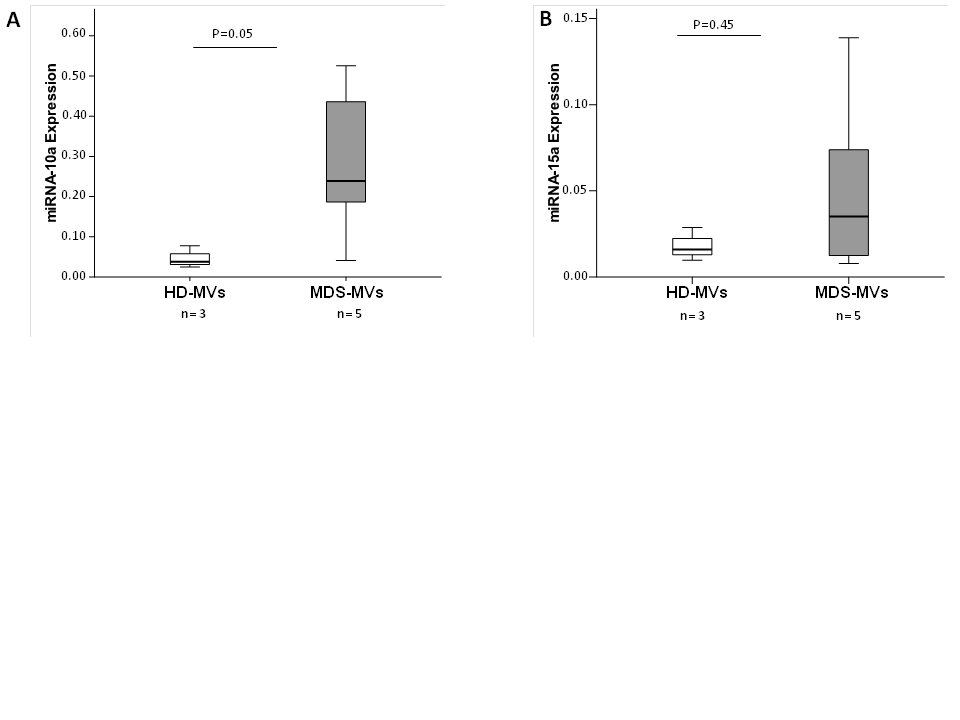

Supplement: S6 Fig — Results expressed as median. (TIF) [file pone.0146722.s006.tif]

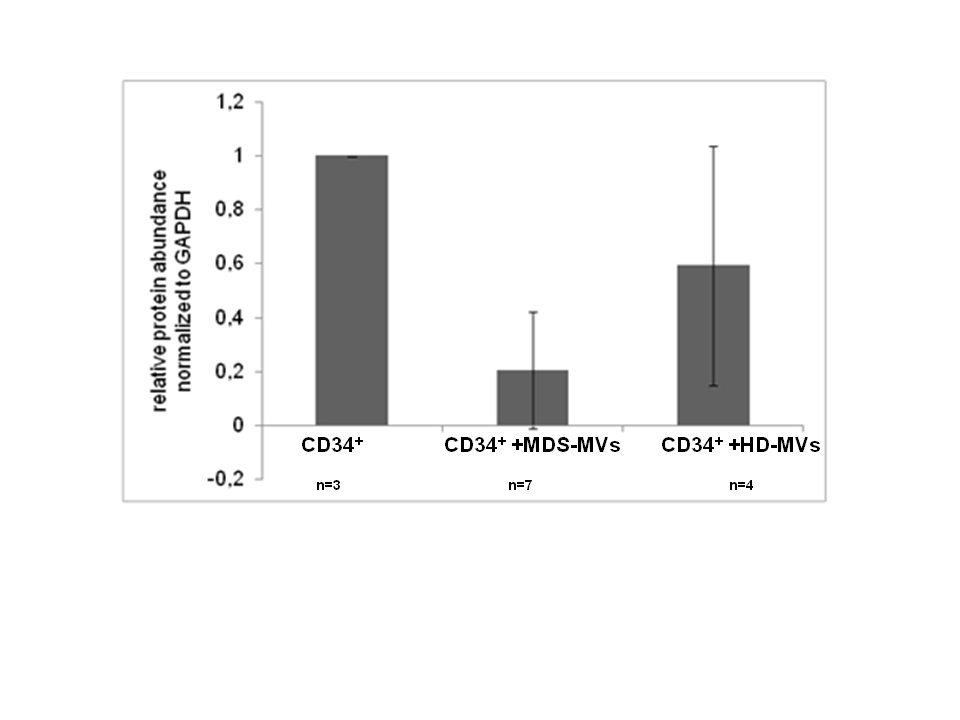

Supplement: S7 Fig — **p<0.01 as assessed by t-test student. (TIF) [file pone.0146722.s007.tif]
