# Supplementary material for: Microvesicles from Mesenchymal Stromal Cells Are Involved in HPC-Microenvironment Crosstalk in Myelodysplastic Patients
Source: PLoS One. 2016 Feb 2;11(2):e0146722. doi: 10.1371/journal.pone.0146722 (PMC4737489; doi:10.1371/journal.pone.0146722)
Supplement: S1 Methods — (DOCX) [file pone.0146722.s008.docx]

**Supplementary Methods**

**Analysis of microRNA differential expression in microvesicles with TaqMan qPCR arrays**

It has been shown that expression of snoRNAs, routinely applied in normalization protocols of real-time PCR following manufacturer’s instructions, present different expression trends to up- or down-regulation in cancer samples (Gee et al. 2011: *The small-nucleolar RNAs commonly used for microRNA normalisation correlate with tumour pathology and prognosis.* British Journal of Cancer, 104, 1168–1177. doi:10.1038/sj.bjc.6606076). This fact would imply a potential bias being introduced in the results of differential expression form Delta-normalized Ct values. Considering that, we have undertaken an analysis of the snoRNA standard controls, and also an extra normalization protocol using different PCR normalization controls.

We have observed in our data that endogenous controls were highly expressed and did not present any trend to increase in MDS samples respect to the healthy donors. The means between sample groups presented no significant difference, as demonstrated by the t-tests performed (which also gave the highest p-values to these control probes). A table of descriptive statistics and t-tests outcome for snoRNA-control variables is shown here:

| **Control probe** | **Raw mean HD** | **Raw mean MDS** | **Mean dCt HD** | **Mean dCt MDS** | **ddCt** | **FC = 2e-ddCt** | **Log10(FC)** | **t.test** | **p.value** |
| --- | --- | --- | --- | --- | --- | --- | --- | --- | --- |
| U6 snRNA | 19.55 | 21.90 | -0.90 | -1.01 | -0.111 | 1.080 | 0.033 | -0.122 | 9.04E-01 |
| U6 snRNA | 19.51 | 21.95 | -0.64 | -0.54 | 0.098 | 0.934 | -0.029 | 0.111 | 9.13E-01 |
| RNU44 | 29.23 | 31.37 | 9.08 | 8.88 | -0.202 | 1.150 | 0.061 | -0.091 | 9.29E-01 |
| RNU48 | 23.01 | 25.36 | 2.86 | 2.87 | 0.015 | 0.990 | -0.004 | 0.015 | 9.88E-01 |
| U6 snRNA | 19.42 | 21.75 | -0.73 | -0.74 | -0.009 | 1.006 | 0.003 | -0.010 | 9.93E-01 |
| U6 snRNA | 19.25 | 21.48 | -0.60 | -0.59 | 0.007 | 0.995 | -0.002 | 0.008 | 9.94E-01 |

We normalized the data again applying a new pool of 5 controls, shown to have stability of expression along our samples. The pool applied in this case included: hsa-miR-16, hsa-miR-222, hsa-let-7b, hsa-let-7e and RNU48.

|  | **Raw Ct HD mean** | **Raw Ct MDS mean** |
| --- | --- | --- |
| hsa-miR-222 | 20.05 | 21.22 |
| hsa-let-7e | 23.26 | 24.65 |
| hsa-miR-16 | 23.48 | 24.74 |
| hsa-let-7b | 23.82 | 25.55 |

Barely different results were observed as shown here:

1. Scatter plot of the normalization effect reproduces the trend to the global up-regulation of miRNAs. **Left**: snoRNAs normalization. **Right**: pool 5 of non-standard stably expressed miRNAs.


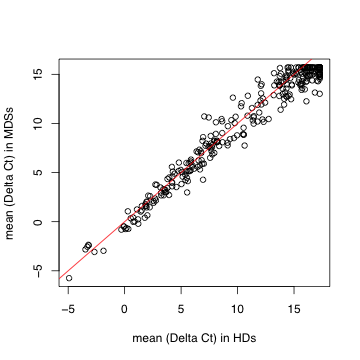


2) The top table of differentially expressed miRNAs is also reproduced. Although, this analysis yielded only the first 14 miRNAs, when ordered by p-values.

**T.test:** Outcome of Student’s tests between MDS patients and healthy donors (HD).

**ddCt or ΔΔCt**: mean ( ΔCt = microRNA_MDS_ ‐ mean (control_MDS_)) ‐ mean (ΔCt = microRNA_HD_ ‐ mean (control_HD_))

**FC = 2^-ddCt^**: Fold Change, is the abundance relative quantification of each microRNA.
