# Supplementary material for: Microvesicles from Mesenchymal Stromal Cells Are Involved in HPC-Microenvironment Crosstalk in Myelodysplastic Patients
Source: PLoS One. 2016 Feb 2;11(2):e0146722. doi: 10.1371/journal.pone.0146722 (PMC4737489; doi:10.1371/journal.pone.0146722)
Supplement: S1 Table — (DOCX) [file pone.0146722.s009.docx]

**Table suplementary:** patients included into all studies

| Patients | | FC | | | TEM | | | | | | IF | | | WB | | | Arrays | | | PCR miRNAs | | | PCR  Gene | | | Cell Viability | | | CFU |  |  |
| --- | --- | --- | --- | --- | --- | --- | --- | --- | --- | --- | --- | --- | --- | --- | --- | --- | --- | --- | --- | --- | --- | --- | --- | --- | --- | --- | --- | --- | --- | --- | --- |
| 1. RCMD | | X |  | | | | | |  | | | |  | | |  | | |  | | |  | | |  | | |  | | | |
| 2. RCMD | |  | | | |  | | |  | | | |  | | | X | | |  | | |  | | |  | | |  | | | |
| 3. 5q- | | X | | | |  | | |  | | | |  | | | X | | |  | | |  | | |  | | |  | | | |
| 4. 5q- | | X | | | |  | | |  | | | |  | | | X | | |  | | |  | | |  | | |  | | | |
| 5. 5q- | |  | | | |  | | |  | | | |  | | | X | | |  | | |  | | |  | | |  | | | |
| 6. RARS | |  | | | |  | | |  | | | |  | | | X | | |  | | |  | | |  | | |  | | | |
| 7. 5q- | |  | | | |  | | |  | | | |  | | | X | | |  | | | X | | |  | | |  | | | |
| 8. RCMD |  | | |  | | | |  | | | |  | | | X | | |  | | |  | | |  | | |  | | | |  |
| 9. RCUD |  | | |  | | | |  | | | |  | | | X | | |  | | |  | | |  | | |  | | | |  |
| 10. RCMD |  | | | X | | | | | | X | |  | | |  | | |  | | |  | | |  | | |  | | | |  |
| 11. RCMD | X | | | X | | | | | |  | |  | | |  | | |  | | |  | | |  | | |  | | | |  |
| 12. RCMD-RS | X | | | | | |  |  | | | |  | | |  | | |  | | |  | | |  | | |  | | | |  |
| 13. RCUD |  | | |  | | | |  | | | |  | | |  | | |  | | | X | | |  | | |  | | | |  |
| 14. RCMD | X | | | X | | | | | |  | |  | | |  | | | X | | |  | | | X | | |  | | | |  |
| 15. RCMD | X | | | X | | | | | |  | |  | | |  | | |  | | | X | | | X(x3) | | |  | | | |  |
| 16. RCMD |  | | |  | | | | | |  | |  | | |  | | | X | | |  | | | X(x2) | | |  | | | |  |
| 17. RCMD |  | | |  | | | |  | | | |  | | |  | | | X | | | X | | | X(X4) | | |  | | | |  |
| 18. RCMD | X | | |  | | | |  | | | |  | | |  | | |  | | |  | | |  | | | X | | | |  |
| 19. AREB-1 |  | | |  | | | |  | | | |  | | |  | | | X | | | X | | |  | | |  | | | |  |
| 20. RCMD |  | | |  | | | |  | | | |  | | |  | | | X | | |  | | |  | | | X | | | |  |
| 21. RCMD |  | | |  | | | |  | | | |  | | |  | | |  | | |  | | |  | | | X | | | |  |
| 22. RCMD |  | | |  | | | |  | | | |  | | |  | | |  | | |  | | |  | | |  | | | |  |
| 23. RA |  | | |  | | | |  | | | |  | | |  | | | X | | | X | | |  | | | X | | | |  |
| 24. RCUD |  | | |  | | | |  | | | |  | | |  | | | X | | |  | | |  | | | X | | | |  |
| 25. RCMD |  | | |  | | | | X | | | | X | | |  | | |  | | | X | | |  | | | X | | | |  |
| 26. 5q- | X | | |  | | | |  | | | | X | | |  | | |  | | |  | | |  | | |  | | | |  |
| 27. RCMD |  | | |  | | | |  | | | | X | | |  | | |  | | | X | | |  | | |  | | | |  |
| 28. RCMD-RS | X | | |  | | | |  | | | | X | | |  | | |  | | | X | | |  | | |  | | | |  |
| 29. RCMD |  | | |  | | | |  | | | | X | | |  | | |  | | |  | | |  | | |  | | | |  |
| 30. RCMD |  | | |  | | | |  | | | | X | | |  | | |  | | |  | | |  | | |  | | | |  |
| TOTAL | 10 | | | 4 | | | | 2 | | | | 6 | | | 8 | | | 7 | | | 9 | | | 10 | | | 6 | | | |  |
