# Supplementary material for: Microvesicles from Mesenchymal Stromal Cells Are Involved in HPC-Microenvironment Crosstalk in Myelodysplastic Patients
Source: PLoS One. 2016 Feb 2;11(2):e0146722. doi: 10.1371/journal.pone.0146722 (PMC4737489; doi:10.1371/journal.pone.0146722)
Supplement: S2 Table — (DOCX) [file pone.0146722.s010.docx]

**Supplementary Table 2:** ***14 microRNAs differentially expressed*** *when normalized with a different selection of control features (ordered by p-values)*

**t-test:** Outcome of Student’s tests between MDS patients and healthy donors (HD).

**ddCt or ΔΔCt**: mean ( ΔCt = microRNA_MDS_ ‐ mean (control_MDS_)) ‐ mean (ΔCt = microRNA_HD_ ‐ mean (control_HD_))

**FC = 2^-ddCt^**: Fold Change, is the abundance relative quantification of each microRNA.
