# Supplementary material for: Microvesicles from Mesenchymal Stromal Cells Are Involved in HPC-Microenvironment Crosstalk in Myelodysplastic Patients
Source: PLoS One. 2016 Feb 2;11(2):e0146722. doi: 10.1371/journal.pone.0146722 (PMC4737489; doi:10.1371/journal.pone.0146722)
Supplement: S3 Table — (DOCX) [file pone.0146722.s011.docx]

| CD34^+^ cells#1 301 colonies | CD34^+^ cells + HD-MVs#1 368 colonies | CD34^+^ cells + MDS-MVs#1 628 colonies |
| --- | --- | --- |
|  | CD34^+^ cells + HD-MVs#2 520 colonies | CD34^+^ cells + MDS-MVs#2 670 colonies |
| CD34^+^ cells#2 876 colonies |  |  |
|  | CD34^+^ cells + HD-MVs#3 906 colonies | CD34^+^ cells + MDS-MVs#3 1046 colonies |
|  | CD34^+^ cells + HD-MVs#4 978 colonies | CD34^+^ cells + MDS-MVs#4 1192 colonies |
|  | CD34^+^ cells + HD-MVs#5 968 colonies | CD34^+^ cells + MDS-MVs#5 1158 colonies |
|  | CD34^+^ cells + HD-MVs#6 972 colonies | CD34^+^ cells + MDS-MVs#6 1166 colonies |

**Supplementary table 3: Number of CFU-GM/5000 CD34^+^ cells**

**CD34^+^ cells:** *CD34^+^ without MVs;* **CD34^+^ cells+HD-MVs:** *CD34^+^ with MVs of healthy donor;* **CD34^+^ cells+MDS-MVs:** *CD34^+^ with MVs of Myelodysplastic syndrome patient.* **# : sample number.**
